# Exercise training comprising of single 20-s cycle sprints does not provide a sufficient stimulus for improving maximal aerobic capacity in sedentary individuals

**DOI:** 10.1007/s00421-016-3409-8

**Published:** 2016-06-06

**Authors:** P. Songsorn, A. Lambeth-Mansell, J. L. Mair, M. Haggett, B. L. Fitzpatrick, J. Ruffino, A. Holliday, R. S. Metcalfe, N. B. J. Vollaard

**Affiliations:** Department for Health, University of Bath, Bath, BA2 7AY UK; Institute of Sport and Exercise Science, University of Worcester, Worcester, WR2 6AJ UK; School of Sport, Ulster University, Derry, Londonderry, BT48 7JL UK

**Keywords:** ${\dot{\text{V}}\text{O}}_{ 2} { \hbox{max} }$, High-intensity interval training, SIT, Wingate sprint, Sprint interval

## Abstract

**Purpose:**

Sprint interval training (SIT) provides a potent stimulus for improving maximal aerobic capacity (${\dot{\text{V}}\text{O}}_{ 2} { \hbox{max} }$), which is among the strongest markers for future cardiovascular health and premature mortality. Cycling-based SIT protocols involving six or more ‘all-out’ 30-s Wingate sprints per training session improve ${\dot{\text{V}}\text{O}}_{ 2} { \hbox{max} }$, but we have recently demonstrated that similar improvements in ${\dot{\text{V}}\text{O}}_{ 2} { \hbox{max} }$ can be achieved with as few as two 20-s sprints. This suggests that the volume of sprint exercise has limited influence on subsequent training adaptations. Therefore, the aim of the present study was to examine whether a single 20-s cycle sprint per training session can provide a sufficient stimulus for improving ${\dot{\text{V}}\text{O}}_{ 2} { \hbox{max} }$.

**Methods:**

Thirty sedentary or recreationally active participants (10 men/20 women; mean ± SD age: 24 ± 6 years, BMI: 22.6 ± 4.0 kg m^−2^, ${\dot{\text{V}}\text{O}}_{ 2} { \hbox{max} }$: 33 ± 7 mL kg^−1^ min^−1^) were randomised to a training group or a no-intervention control group. Training involved three exercise sessions per week for 4 weeks, consisting of a single 20-s Wingate sprint (no warm-up or cool-down). ${\dot{\text{V}}\text{O}}_{ 2} { \hbox{max} }$ was determined prior to training and 3 days following the final training session.

**Results:**

Mean ${\dot{\text{V}}\text{O}}_{ 2} { \hbox{max} }$ did not significantly change in the training group (2.15 ± 0.62 vs. 2.22 ± 0.64 L min^−1^) or the control group (2.07 ± 0.69 vs. 2.08 ± 0.68 L min^−1^; effect of time: *P* = 0.17; group × time interaction effect: *P* = 0.26).

**Conclusion:**

Although we have previously demonstrated that regularly performing two repeated 20-s ‘all-out’ cycle sprints provides a sufficient training stimulus for a robust increase in ${\dot{\text{V}}\text{O}}_{ 2} { \hbox{max} }$, our present study suggests that this is not the case when training sessions are limited to a single sprint.

## Introduction

In cross-sectional studies, maximal aerobic capacity (${\dot{\text{V}}\text{O}}_{ 2} { \hbox{max} }$) is one of the strongest prognostic markers for future cardiovascular health and premature death (Myers et al. 2002; Keteyian et al. 2008). Moreover, improving ${\dot{\text{V}}\text{O}}_{ 2} { \hbox{max} }$ is associated with substantial reductions in the risk for mortality during follow-up in longitudinal studies (Blair et al. 1995; Lee et al. 2011). Regular physical activity (PA) is the only feasible means of improving absolute ${\dot{\text{V}}\text{O}}_{ 2} { \hbox{max} }$, but the association between PA levels and mortality disappears after adjustment for ${\dot{\text{V}}\text{O}}_{ 2} { \hbox{max} }$ (Lee et al. 2011), suggesting that a high ${\dot{\text{V}}\text{O}}_{ 2} { \hbox{max} }$ is more important than high PA levels. Thus, it has been recommended that besides encouraging reductions in sedentary time and increases in overall PA, improving ${\dot{\text{V}}\text{O}}_{ 2} { \hbox{max} }$ should also be a key public health message (Lee et al. 2011; Bouchard et al. 2015).

PA guidelines based on moderate-intensity aerobic exercise have been consistently promoted for over two decades (Pate et al. 1995; Garber et al. 2011), but the adherence to these recommendations remains poor in the general population (Hallal et al. 2012). To address the commonly reported barrier of lack of time (Korkiakangas et al. 2009), submaximal high-intensity interval training (HIIT) and supramaximal sprint interval training (SIT) have been proposed as time-efficient alternative/adjunct exercise strategies (Gillen and Gibala 2014). A common type of SIT protocol consists of 4–10 repeated 30-s ‘all-out’ Wingate sprints, thus resulting in just 2–5 min of high-intensity exercise per session (Weston et al. 2014). Such protocols have been shown to provide a robust increase in ${\dot{\text{V}}\text{O}}_{ 2} { \hbox{max} }$, superior to that following aerobic exercise training (Burgomaster et al. 2008; Bailey et al. 2009; Macpherson et al. 2011; Sandvei et al. 2012; Nalcakan 2014; Milanovic et al. 2015). However, the low volume of high-intensity exercise does not necessarily result in a time-efficient exercise intervention *per se*, as the need for recovery periods in between sprints generally results in a total training time commitment in excess of 30 min per session (Gillen and Gibala 2014).

Because the mechanisms by which SIT improves ${\dot{\text{V}}\text{O}}_{ 2} { \hbox{max} }$ are poorly understood, it also remains unknown how the training stimulus can be optimised in order to achieve either the largest increases in ${\dot{\text{V}}\text{O}}_{ 2} { \hbox{max} }$, or a set increase using the smallest amount of time and effort. However, recent evidence suggests that Wingate-based SIT protocols can be made shorter and less strenuous while retaining the positive effect on ${\dot{\text{V}}\text{O}}_{ 2} { \hbox{max} }$. Two studies have directly compared the effects of reducing sprint duration from 30 s to either 10 s (Hazell et al. 2010) or 15 s (Zelt et al. 2014), and neither study observed a lower increase in ${\dot{\text{V}}\text{O}}_{ 2} { \hbox{max} }$ with the shorter sprint duration. Furthermore, four training studies have examined the effect on ${\dot{\text{V}}\text{O}}_{ 2} { \hbox{max} }$ of SIT protocols incorporating fewer than four supramaximal sprints per session. SIT protocols consisting of three 20-s sprints (Gillen et al. 2014) or three 30-s sprints (Allemeier et al. 1994; Ijichi et al. 2015) were reported to increase ${\dot{\text{V}}\text{O}}_{ 2} { \hbox{max} }$ by 12–14 %. Moreover, in our lab we observed a mean increase of 14 % (Metcalfe et al. 2012) following a SIT protocol with just two 20-s all-out sprints. Changes of such magnitude favourably compare with more strenuous SIT protocols: recent meta-analyses have reported a range of improvements in ${\dot{\text{V}}\text{O}}_{ 2} { \hbox{max} }$ of 3–14 % for SIT studies involving 4–10 repeated Wingate sprints per session (Sloth et al. 2013; Gist et al. 2014; Weston et al. 2014).

The fact that performing fewer and/or shorter supramaximal sprints is sufficient for improving ${\dot{\text{V}}\text{O}}_{ 2} { \hbox{max} }$ suggests that the total volume of high-intensity exercise is not a key determinant of the training stimulus. Conversely, it is plausible to hypothesise that the training stimulus resides predominantly within the first of repeated sprints. If this is indeed the case, then an exercise training protocol consisting of a single supramaximal sprint per session should be sufficient to increase ${\dot{\text{V}}\text{O}}_{ 2} { \hbox{max} }$. There is some mechanistic support for this hypothesis: the signalling molecule AMPK, which is deemed important for aerobic adaptations (Gibala et al. 2012), is regulated by glycogen availability (McBride et al. 2009). It has been shown that glycogen depletion during repeated supramaximal sprints is limited to the first sprint (Parolin et al. 1999), and AMPK activation has been observed in response to a single Wingate sprint (Guerra et al. 2010; Fuentes et al. 2012).

Considering the strong association between ${\dot{\text{V}}\text{O}}_{ 2} { \hbox{max} }$ and health, and the fact that lack of time is consistently reported as an important barrier to performing sufficient exercise, there is a need to identify the lowest volume of exercise effective at modifying ${\dot{\text{V}}\text{O}}_{ 2} { \hbox{max} }$. Thus, the aim of the present randomised controlled trial was to determine whether regularly performing a single 20-s ‘all-out’ cycle sprint provides a sufficient training stimulus for improving ${\dot{\text{V}}\text{O}}_{ 2} { \hbox{max} }$ in sedentary or recreationally active individuals.

## Methods

### Compliance with ethical standards

The study was approved by the local University Ethics committees (reference: EP 14/15 87/FC272014-2015), and conformed to the standards set forth in the latest revision of the Declaration of Helsinki. The study protocol was fully explained to all participants in written and verbal form before they were asked to provide written consent.

### Participants

Thirty apparently healthy, sedentary or recreationally active participants (10 men/20 women; mean ± SD age 24 ± 6 years, BMI 22.6 ± 4.0 kg m^−2^, ${\dot{\text{V}}\text{O}}_{ 2} { \hbox{max} }$ 33 ± 7 mL kg^−1^ min^−1^) were recruited at three sites in the UK (Bath, Worcester, Derry/Londonderry) and randomised into a training group (*n* = 15; 5 men) and a control group (*n* = 15; 5 men). Exclusion criteria were classification as highly physically active according to the International physical activity questionnaire [IPAQ (Craig et al. 2003)], contraindications to exercise as determined using a standard physical activity readiness questionnaire [PAR-Q (Thomas et al. 1992)], clinically significant hypertension (>140/90 mm Hg), or resting heart rate ≥100 bpm. Using the IPAQ, the activity level of 16 participants was categorised as ‘low’, and the activity level of the remaining 14 participants as ‘moderate’.

### Experimental procedures

An incremental cycling test to exhaustion was performed on an electronically braked ergometer to determine ${\dot{\text{V}}\text{O}}_{ 2} { \hbox{max} }$ (Excalibur Sport/Corival, Lode, Groningen, The Netherlands). Participants were asked not to perform strenuous exercise or consume caffeine or alcohol the day before and prior to the test, and to drink half a litre of water the morning of the testing day. Participants completed a 2-min warm-up at 50 W after which the intensity was increased by 1 W every 3 s until volitional exhaustion despite verbal encouragement. Oxygen uptake (V̇O_2_) was determined throughout the test using an online gas analyser (TrueOne 2400, Parvo Medics, Sandy, UT, US; COSMED Quark CPET, Rome, Italy; Oxycon Pro, Jaeger, Wurzburg, Germany) to determine ${\dot{\text{V}}\text{O}}_{ 2} { \hbox{max} }$ as the highest value for a 15-breath rolling average. Values for ${\dot{\text{V}}\text{O}}_{ 2} { \hbox{max} }$ were accepted if two or more of the following criteria were met: (1) volitional exhaustion, (2) RER > 1.15, and (3) maximal heart rate within 10 beats of the age-predicted maximum (i.e., 220 age). This was the case for all participants, except for one control participant who was excluded from the data analysis (inclusion of this participant did not alter the statistical results).

Following the ${\dot{\text{V}}\text{O}}_{ 2} { \hbox{max} }$ test, participants in the training group started a 4-week training programme consisting of three training sessions per week. Training sessions involved a single 20-s ‘all-out’ adapted Wingate sprint against a resistance equivalent to 7.5 % of the participant’s pre-training body mass. Participants were asked to achieve the highest pedal frequency possible during a ~1–2 s unloaded lead-in prior to applying the full resistance. Participants then received strong verbal encouragement to maintain the highest pedal frequency throughout the remaining 18–19 s. To allow us to specifically investigate the effects of the 20-s sprints, no warm-up or cool-down were performed. To be included in the final data analyses, participants could not miss more than two training sessions, two training sessions within the final week, or the final training session. No participants failed to meet these criteria. Participants allocated to the control group were asked to maintain their usual physical activity patterns for the duration of the study. All participants were asked not to change their current eating behaviour.

Acute exercise-induced changes in plasma volume were determined during the first and last training sessions. After at least 15 min of seated rest, a finger-prick blood sample was taken and directly analysed for haemoglobin (Hb) levels (HemoCue, Crawley, UK). A sample for determination of haematocrit (Hct) was stored for analysis after all subsequent samples had been collected (Haematospin 1300; Hawksley & Son Ltd, Lancing, UK). A second sample was taken directly after completion of the sprint, with further samples taken at rest in a seated position at 3, 10 and 30 min following the end of the sprint. Plasma volume changes were calculated as described by Dill and Costill (1974). Peak (PPO), end (EPO), and mean power output (MPO), as well as peak heart rate, were recorded during the 3rd and 12th training sessions. rating of perceived exertion [RPE (Borg 1970)] was determined directly after the 3rd, 6, 9 and 12th training sessions.

A second ${\dot{\text{V}}\text{O}}_{ 2} { \hbox{max} }$ test was performed 3 days after the final training session, at a similar time as the baseline test and following identical procedures. Participants in the control group performed their second ${\dot{\text{V}}\text{O}}_{ 2} { \hbox{max} }$ test after a similar duration compared to participants in the training group. For the day before testing and on the testing day itself, participants were asked to follow a diet similar to that for the baseline test.

### Statistical analysis

Data are presented as mean ± SD. Based on a coefficient of variation of the ${\dot{\text{V}}\text{O}}_{ 2} { \hbox{max} }$ test protocol of 4 %, it was calculated that 14 participants were needed in each group in order to be able to detect a difference in the change in ${\dot{\text{V}}\text{O}}_{ 2} { \hbox{max} }$ of 5 % between the training group and the control group, with a power of 90 % and *α* = 0.05. Two-way mixed model ANOVAs (group × time) were performed to determine differences in the change in Wmax and ${\dot{\text{V}}\text{O}}_{ 2} { \hbox{max} }$ from baseline to post-intervention between the training group and the control group. Two-way repeated measures ANOVAs (training session × time) were used to assess the effect of acute exercise on plasma volume change. Differences in peak HR, PPO, MPO and EPO between the 3rd and 12th training sessions were determined using paired-sample *t* tests. Alpha was set at 0.05.

## Results

There were no significant differences in mean baseline characteristics between participants in the control group and the training group (Control—age: 23 ± 5 years, BMI: 22.4 ± 3.5 kg m^−2^, ${\dot{\text{V}}\text{O}}_{ 2} { \hbox{max} }$: 32 ± 6 mL kg min^−1^; Training—age: 24 ± 6 years, BMI: 22.9 ± 4.5 kg m^−2^, ${\dot{\text{V}}\text{O}}_{ 2} { \hbox{max} }$: 34 ± 8 mL kg min^−1^). Body mass did not change from baseline to reassessment in the training group (63.6 ± 15.6 vs. 63.9 ± 14.9 kg) or in the control group (64.4 ± 12.8 vs. 64.4 ± 12.8 kg). Of the fifteen participants in the training group, twelve completed all 12 training sessions, two completed 11 sessions, and one completed 10 sessions, resulting in an overall mean adherence of 98 %. Characteristics of the training sessions are provided in Table 1. Peak and mean power output were not significantly different between the third and twelfth training sessions, but end power output was increased by 9 % in the 12th compared to the 3rd session (*P* = 0.03). Peak heart rate reached 90 ± 11 and 91 ± 4 % of HRmax during the 3rd and 12th training sessions, respectively. Plasma volume was significantly reduced throughout the post-exercise period (*P* = 0.02), with no difference between the post-exercise time-points or between sessions 1 and 12 (Table 1).

**Table 1:** Training characteristics (*n* = 15)

	Training session				
	1	3	6	9	12
PPO (W kg^−1^)	–	8.6 ± 1.7	–	–	8.9 ± 1.8
MPO (W kg^−1^)	–	7.0 ± 1.4	–	–	7.1 ± 1.4
EPO (W kg^−1^)	–	5.3 ± 1.5	–	–	5.8 ± 1.4*
HRpeak (% of HRmax)	–	90 ± 11	–	–	91 ± 4
RPE	–	16 ± 2	14 ± 2	14 ± 2	14 ± 2
ΔPV at *t* = 0 (% change from pre-exercise)	−8 ± 7	–	–	–	−8 ± 7
ΔPV at *t* = 3 (% change from pre-exercise)	−10 ± 6	–	–	–	−10 ± 7
ΔPV at *t* = 10 (% change from pre-exercise)	−10 ± 6	–	–	–	−8 ± 6
ΔPV at *t* = 30 (% change from pre-exercise)	−8 ± 5	–	–	–	−1 ± 5

Values shown are mean ± SD

*PPO* peak power output, *MPO* mean power output, *EPO* end power output, *RPE* rating of perceived exertion, Δ*PV* plasma volume change

* *P* < 0.05 for the difference between the 3rd and 12th training session

**Fig. 1:**
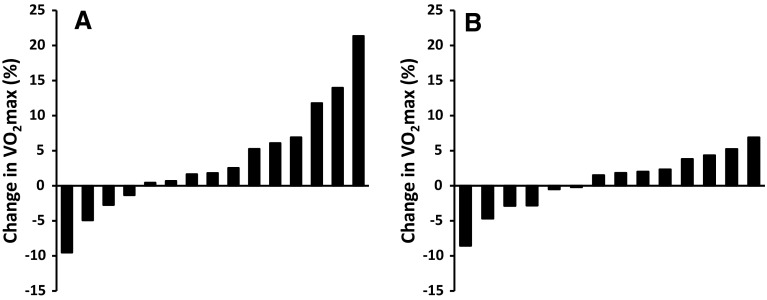
Individual changes in ${\dot{\text{V}}\text{O}}_{ 2} { \hbox{max} }$ for the training group (**a**) and the control group (**b**)

Maximal power output (Wmax) was increased in the training group (185 ± 50 vs. 195 ± 50 W) compared to the control group (180 ± 48 vs. 174 ± 43 W; group × time interaction effect: *P* = 0.001). However, mean ${\dot{\text{V}}\text{O}}_{ 2} { \hbox{max} }$ did not significantly change from baseline in the training group (2.15 ± 0.62 vs. 2.22 ± 0.64 L min^−1^) or the control group (2.07 ± 0.69 vs. 2.08 ± 0.68 L min^−1^; effect of time: *P* = 0.17; group × time interaction effect: *P* = 0.26). There were no significant correlations between the change in ${\dot{\text{V}}\text{O}}_{ 2} { \hbox{max} }$ and either physical activity levels as measured using the International physical activity questionnaire (*R*^2^ = 0.06), or baseline ${\dot{\text{V}}\text{O}}_{ 2} { \hbox{max} }$ (*R*^2^ = 0.00). Interindividual variability in the change in ${\dot{\text{V}}\text{O}}_{ 2} { \hbox{max} }$ was larger in the training group (range −10 to +21 %) compared to the control group (−9 to +7 %; Fig. 1).

## Discussion

Considering the strong association between improved ${\dot{\text{V}}\text{O}}_{ 2} { \hbox{max} }$ and reduced all-cause/CVD mortality, it is important for studies to identify the most effective training modalities for improving ${\dot{\text{V}}\text{O}}_{ 2} { \hbox{max} }$ (Weston et al. 2014). While strategies aimed at offering the greatest improvements should be a priority, considering the commonly reported barrier of lack of time (Korkiakangas et al. 2009) there is also a need for studies examining protocols that enable improved ${\dot{\text{V}}\text{O}}_{ 2} { \hbox{max} }$ with a minimal time commitment. Although our previous studies have demonstrated that regularly performing just two repeated 20-s all-out cycle sprints causes a robust increase in ${\dot{\text{V}}\text{O}}_{ 2} { \hbox{max} }$ (Metcalfe et al. 2012), in the present study we have established that a single sprint does not provide a sufficient training stimulus.

Considering the proclaimed aim for SIT to be a time-efficient alternative exercise intervention, it is surprising that little attention has been given to identifying the lowest volume of high-intensity exercise associated with beneficial adaptations. The design of commonly studied SIT protocols has not been evidence based, but instead appears to have utilised an arbitrary number of sprint repetitions, perhaps at most guided by finding a balance between the largest total volume of high-intensity exercise and a manageable total exercise session duration. There is, however, no evidence that a larger number of sprint repetitions or a greater total volume of sprint exercise will lead to superior cardiovascular or metabolic adaptations. In our lab we have shown that very low volumes of supramaximal exercise [40 s per exercise session; REHIT (Metcalfe et al. 2012)] are associated with improvements in ${\dot{\text{V}}\text{O}}_{ 2} { \hbox{max} }$ typically seen with much higher volumes [≥180 s per session (Weston et al. 2014; Milanovic et al. 2015)]. In this light, the difference in effectiveness between the present protocol (1 × 20-s sprint, ~3 % mean increase in ${\dot{\text{V}}\text{O}}_{ 2} { \hbox{max} }$) and our REHIT protocol [2 × 20-s sprints; 14 % mean increase in ${\dot{\text{V}}\text{O}}_{ 2} { \hbox{max} }$ (Metcalfe et al. 2012)] is striking.

One potential explanation is that the present study used an intervention period of 4 weeks, whereas in our previous study the intervention lasted 6 weeks. However, adaptations to SIT protocols tend to occur very rapidly, and other studies have demonstrated significant increases in ${\dot{\text{V}}\text{O}}_{ 2} { \hbox{max} }$ after as little as two weeks of training (Hazell et al. 2010; Whyte et al. 2010). Furthermore, Ijichi et al. (2015) observed a 14 % improvement in ${\dot{\text{V}}\text{O}}_{ 2} { \hbox{max} }$ following a 4-week SIT intervention with three 30-s sprints per training session, suggesting that large improvements in ${\dot{\text{V}}\text{O}}_{ 2} { \hbox{max} }$ are feasible with a small volume of high-intensity exercise within a short period of time. Moreover, data reported by Burgomaster et al. (2008) suggest no further increase in ${\dot{\text{V}}\text{O}}_{ 2} { \hbox{max} }$ after 3 out of 6 weeks of SIT, and the meta-analysis by Weston et al. (2014) suggests that training duration is not a significant modifier of the increase in ${\dot{\text{V}}\text{O}}_{ 2} { \hbox{max} }$ with SIT: a threefold increase in the number of sessions has an unclear effect on the change in ${\dot{\text{V}}\text{O}}_{ 2} { \hbox{max} }$ of −0.3 %.

It could also be argued that doubling the volume of sprint exercise per session from 20 to 40 s may provide a substantial additional training stimulus, thus explaining the superior increase in ${\dot{\text{V}}\text{O}}_{ 2} { \hbox{max} }$ with repeated sprints. However, there are currently no indications that further increasing the number of sprint repetitions to more than two further enhances the increase in ${\dot{\text{V}}\text{O}}_{ 2} { \hbox{max} }$, with the majority of studies examining 6–10 repeated Wingate sprints reporting increases in ${\dot{\text{V}}\text{O}}_{ 2} { \hbox{max} }$ similar to—or lower than—those with two repeated sprints (McKenna et al. 1997; MacDougall et al. 1998; Burgomaster et al. 2008; Hazell et al. 2010; Whyte et al. 2010). If the low total sprint volume was to explain the lack of effect in the present study, then it remains unclear why there is no evidence for an association between larger sprint volumes and enhanced improvements in ${\dot{\text{V}}\text{O}}_{ 2} { \hbox{max} }$. An alternative explanation could be that the initial sprint of a SIT session provides a ‘priming’ effect which then allows subsequent sprints to be effective, but this remains speculation at this point.

While a training programme incorporating single 20-s sprints may not be of use to improve aerobic capacity in the general population, the divergent responses to training protocols consisting of one vs. two sprints may provide an opportunity to help elucidate the molecular mechanisms through which SIT improves ${\dot{\text{V}}\text{O}}_{ 2} { \hbox{max} }$. One problem in studying mechanisms is that cause–effect relations cannot be established by correlating acute effects of exercise to chronic adaptations following training. However, by contrasting the acute effects of exercise protocols that are similar, but with divergent training effects, it may be possible to provide evidence against specific proposed mechanisms. It is clear that if a specific acute response is observed following both a single 20-s sprint (insufficient stimulus for improving ${\dot{\text{V}}\text{O}}_{ 2} { \hbox{max} }$) and two repeated 20-s sprints (sufficient stimulus), then this response by itself cannot be responsible for causing the training adaptation(s). Future studies are needed to assess the potential use of this approach. Furthermore, future large studies should further examine the impact of technical error (measurement error and day-to-day biological variability) on the occurrence of low response to training protocols. The impact of this issue on the results in the current study are unclear, and only studies with a large sample size can sufficiently address this issue (Ross et al. 2015).

Some limitations of the present study mean that the results should be treated with caution. Firstly, we did not include a third arm to the RCT to directly compare the single-sprint protocol to a repeated-sprint protocol or to a protocol incorporating 30-s Wingate sprints. Future studies should determine whether performing single sprints of a longer duration may improve ${\dot{\text{V}}\text{O}}_{ 2} { \hbox{max} }$. Secondly, the interindividual variability in response shown in Fig. 1 suggests that the single-sprint training protocol may have been effective for some participants, with 20 % of the participants in the training group showing an increase in ${\dot{\text{V}}\text{O}}_{ 2} { \hbox{max} }$ of >10 %. Thus, it may be that the mean increase in ${\dot{\text{V}}\text{O}}_{ 2} { \hbox{max} }$ in response to single-sprint training in a larger sample would reach significance. However, the power of our study was sufficient to be able to detect a difference in the change in ${\dot{\text{V}}\text{O}}_{ 2} { \hbox{max} }$ of 5 % between the control group and the training group, and smaller changes would not justify using a single-sprint training protocol instead of the more effective REHIT protocol or other more strenuous SIT protocols.

In conclusion, our present study suggests that regularly performing a single 20-s ‘all-out’ cycle sprint does not provide a sufficient training stimulus for increasing ${\dot{\text{V}}\text{O}}_{ 2} { \hbox{max} }$ in low- to moderately active men and women. Thus, the REHIT protocol, consisting of two 20-s cycle sprints within a 10-min exercise session, remains the lowest volume of exercise shown to improve robustly ${\dot{\text{V}}\text{O}}_{ 2} { \hbox{max} }$. Further studies investigating the optimal way to modulate important cardiometabolic risk factors in a time-efficient manner may help improve the health and quality of life in the predominantly insufficiently active general population.

## Abbreviations

AMPK: Adenosine monophosphate-activated protein kinase
ANOVA: Analysis of variance
BMI: Body mass index
CVD: Cardiovascular disease
EPO: End power output
Hb: Haemoglobin
Hct: Haematocrit
HR: Heart rate
IPAQ: International physical activity questionnaire
MPO: Mean power output
PA: Physical activity
PAR-Q: Physical activity readiness questionnaire
PPO: Peak power output
RCT: Randomised controlled trial
REHIT: Reduced-exertion high-intensity interval training
RER: Respiratory exchange ratio
RPE: Rating of perceived exertion
SIT: Sprint interval training
${\dot{\text{V}}\text{O}}_{ 2} { \hbox{max} }$: Maximal aerobic capacity
Wmax: Maximal power output
